# Perceptions and opinions on the COVID-19 pandemic in flanders, belgium: Data from a three-wave longitudinal study

**DOI:** 10.1016/j.dib.2020.106060

**Published:** 2020-07-23

**Authors:** David De Coninck, Leen d'Haenens, Koen Matthijs

**Affiliations:** a Centre for Sociological Research, KU Leuven, Oude Markt 13, Leuven, Belgium; b Institute for Media Studies, KU Leuven, Oude Markt 13, Leuven, Belgium

**Keywords:** Covid-19, Coronavirus, Belgium, Longitudinal, Attitudes, Public health, Pandemic, crisis

## Abstract

During the COVID-19 pandemic, people have become increasingly fearful of the disease as death tolls rise, while governments attempt to combat it by installing restrictive measures. News media play a vital role as they are the main sources from which people gather information regarding the disease and the public health measures. The present longitudinal data reflect a bird's eye view of people's fears towards getting ill, their news media consumption, and their attitudes regarding the (Belgian) government's handling of the COVID-19 crisis. Data were collected at three key moments in the pandemic among adults in Flanders, Belgium: in the middle of March (when the first restrictive measures went into effect; *N* = 1,000), early April (as hospital admissions and death toll peaked; *N* = 870), and at the end of May and beginning of June (as several measures were lifted or relaxed; *N* = 768). With only 23.2% drop-out across the three waves, these data may be of interest to researchers who wish to explore dynamics of fear and attitudes towards public health measures during this particularly challenging time.

Specifications Table

**Table:** 

Subject	Public Health and Health Policy; Social Sciences
Specific subject area	Attitudes towards public health; Fear of disease; Media consumption
Type of data	Table
How data were acquired	Three-wave online survey among the adult population in Flanders, Belgium
Data format	Raw, analysed, and filtered.
Parameters for data collection	Being over the age of 18 and under the age of 70 and residing in Flanders, Belgium at the time of the study (March through June of 2020).
Description of data collection	We collected the data in cooperation with a Belgian survey agency and selected the methodology for its cost-effectiveness in large-scale and longitudinal research. Respondents received an e-mail asking them to participate in a survey without specifying the subject matter. Data were collected at three key moments in the pandemic among adults in Flanders, Belgium: in the middle of March (when the first restrictive measures went into effect; *N* = 1000), early April (as hospital admissions and death toll peaked; *N* = 870), and at the end of May and beginning of June (as several measures were lifted or relaxed; *N* = 768). The initial response rate was about 35%, the drop-out rate between Wave 1 and Wave 3 was 23.2%.
Data source location	Institution: KU Leuven City/Town/Region: Leuven Country: Belgium
Data accessibility	Repository name: Perceptions and opinions on the COVID-19 pandemic in Flanders, Belgium: Data from a three-wave longitudinal study Data identification number: http://dx.doi.org/10.17632/mhx3p7w3d6.2 Direct URL to data: https://data.mendeley.com/datasets/mhx3p7w3d6/2

## Value of the Data

• The data presented can help researchers to better understand the relationship between perceived vulnerability to disease, news media consumption, and attitudes towards public health measures among adults during the COVID-19 pandemic in Flanders, Belgium.
• Public health researchers can benefit from these data because they highlight how attitudes towards governmental measures differ between individuals, and across time, while media scholars may be particularly interested in the role that news media consumption plays in this regard.
• These data may be used to analyze quarantine strategies and contexts for their social acceptability, determining best ways to apply knowledge about infection prevention and control, and enhance (or develop) an ethical framework for outbreak response.
• The data presented are rare: there is currently very little longitudinal COVID-19 research that combines indicators on fear and attitudes with measures on news media consumption. This allows for new insights in a field which is rapidly evolving, and where researchers and policy makers alike are looking for new insights.

## 1 Data description

The data presented in this article were collected during the COVID-19 pandemic in Flanders, Belgium. The aim of this data collection was to investigate the dynamic interplay between perceived vulnerability to disease, attitudes towards public health measures, and news media consumption among adults aged 18 to 70 at key moments of the crisis in Flanders, Belgium. Data were collected three times in 2020: from March 17 to March 22 (when the first restrictive measures went into effect; *N* = 1000), from April 6 to April 18 (as hospital admissions and the death toll peaked; *N* = 870), and from May 17 to June 5 (as several measures were lifted or relaxed; *N* = 768). The Belgian survey agency we worked with (iVOX) drew the initial sample out of its large-scale panels (150,000 individuals). The response rate was about 32 percent, and responses were gathered from an opt-in online panel that used quotas by gender, age, education, and province to ensure the data were representative for these characteristics in Flanders. Respondents were contacted by e-mail, and the survey was distributed via the polling agency's own survey tool. The survey language was Dutch, the official language in Flanders. Respondents were unable to skip questions, but some questions did have a ‘no answer’-option. Each question in the survey was presented on a different page, and there was no option to return to previous questions and change any answers. Upon completion of the survey, respondents were given a number of virtual points. After collecting a certain amount of points, they were able to purchase gift coupons for restaurant visits, trips, and other activities, from the website of the survey agency. All respondents who recorded partial data were removed by the survey agency prior to delivering the final, fully anonymized, dataset.

For the subsequent waves, only the original 1000 respondents from Wave 1 were contacted in order to ensure that we could investigate intrapersonal changes throughout the crisis and maintain the longitudinal design. The overall drop-out rate was 13% between Wave 1 and Wave 2 and 11.7**%** between Wave 2 and Wave 3, for a total drop-out of 23.2% between Wave 1 and Wave 3. Of the 768 respondents in Wave 3, 742 completed all three waves (96.6%). Table 1 shows the distribution of respondents by several socio-demographic characteristics and per wave, while Table 2 presents mean scores of selected indicators on perceived vulnerability to disease, attitudes towards public health measures, news media consumption, and resilience.

**Table 1:** Socio-demographic characteristics of participants in the three waves (in%, unless otherwise specified).

	Wave 1 (N = 1000)	Wave 2 (N = 870)	Wave 3 (N = 768)
Gender			
Male	49.5	51.3	52.0
Female	50.5	48.7	48.0
Mean age (in years)	47.25	48.49	49.27
Educational attainment			
No degree/Primary degree	2.9	3.2	3.3
Secondary degree	48.7	49.1	50.2
Tertiary degree	48.4	47.6	46.5
Perceived income (average score)^a^	4.07	4.08	4.16
Political ideology (average score)^b^	4.27	4.27	4.31
Living environment			
Large city	10.0	10.0	9.8
Suburbs	17.1	16.6	16.5
Small city	22.5	21.7	22.4
Village	43.7	45.1	45.2
Countryside	6.5	6.4	6.1
Other	0.2	0.2	0
(Work) situation			
Full-time job	49.1	46.2	46.5
Part-time job	12.0	12.1	11.2
Temporarily disabled	1.8	1.8	1.2
Permanently disabled	3.0	3.0	3.3
Student	5.7	5.5	3.6
Houseman/housewife	3.4	3.7	4.0
Unemployed	2.2	2.3	2.7
Retired	24.6	27.0	28.8
Respondent was requested to work from home during COVID-19 crisis			
Yes	33.7	32.2	29.3
No	31.6	27.2	28.6
N/A^c^	34.7	40.6	42.1
Company that respondent worked at closed down due to COVID-19 crisis			
Yes	18.5	18.3	10.4
No	46.8	42.2	47.5
N/A^c^	34.7	39.5	42.1

Note:.

^a^Perceived income was measured by asking respondents about easily they make do with their current income, ranging from 1 = very difficult to 5 = very easily.

^b^Political ideology was measured through the following question: ‘When it comes to politics, people talk about ‘left’ and ‘right’. Where would you place yourself on the scale below, where 1 stands for far left and 7 for far right?’.

^c^Denotes respondents that did not work prior to crisis (disabled people, students, housemen/wives, unemployed people, retirees).

**Table 2:** Mean scores of selected indicators on perceived vulnerability to disease, news media consumption and attitudes towards public health measures.

	Wave 1 (*N* = 1000)	Wave 2 (*N* = 870)	Wave 3 (*N* = 768)
Perceived vulnerability to disease			
It really bothers me when people sneeze without covering their mouths.	6.03	6.20	6.10
If an illness is ‘going around’, I will get it.	3.10	3.02	2.79
I am comfortable sharing a water bottle with a friend.	3.15	2.72	2.77
I do not like to write with a pencil someone else has obviously chewed on.	5.24	5.41	5.47
My past experiences make me believe I am not likely to get sick even when my friends are sick.	3.44	3.40	3.60
I have a history of susceptibility to infectious disease.	2.75	2.61	2.41
I prefer to wash my hands pretty soon after shaking someone's hand.	4.30	4.71	4.62
In general, I am very susceptible to colds, flu and other infectious diseases.	3.45	3.33	3.18
I dislike wearing used clothes because you do not know what the last person who wore it was like.	4.71	4.78	4.66
I am more likely than the people around me to catch an infectious disease.	3.05	2.90	2.78
My hands do not feel dirty after touching money.	4.05	3.92	3.97
I am unlikely to catch a cold, flu or other illness, even if it is ‘going around’.	3.32	3.40	3.57
It does not make me anxious to be around sick people.	3.69	3.43	3.71
My immune system protects me from most illnesses that other people get.	3.73	3.59	3.76
I avoid using public telephones because of the risk that I may catch something from the previous user.	3.27	3.50	3.46
News media consumption			
Public television	3.76	3.54	3.32
Commercial television	3.16	2.97	2.76
Public radio	3.27	3.11	2.96
Commercial radio	2.27	2.19	2.07
Quality newspaper	2.31	2.21	2.08
Popular newspaper	3.18	3.13	3.02
Social media of public news/quality newspaper	2.88	2.66	2.42
Social media of commercial news/popular newspaper	2.86	2.71	2.50
Attitudes towards public health measures			
Measures are necessary to protect population	4.63	4.54	4.07
Fear of economic crisis	4.03	4.15	4.19
Fear of loneliness	2.83	2.61	2.35
Quarantine when feeling sick	4.12	4.05	3.89
Government handles crisis well	3.56	3.57	3.10
Brief Resilience Scale			
I tend to bounce back quickly after hard times	–	–	3.80
I have a hard time making it through stressful events.	–	–	2.57
It does not take me long to recover from a stressful event.	–	–	3.41
It is hard for me to snap back when something bad happens.	–	–	2.92
I usually come through difficult times with little trouble	–	–	3.40
I tend to take a long time to get over set-backs in my life.	–	–	2.59

## 2 Experimental design, materials and methods

Although several population surveys were conducted into attitudes regarding COVID-19 or public health measures, few were longitudinal. An inherent shortcoming of cross-sectional surveys is that they cannot make any causal claims, while longitudinal studies provide more context regarding the complex nature of such relationships. To recapitulate, we developed an online public opinion survey, to be carried out amongst a sample of Flemish residents aged 18 to 70, representative for gender, age, educational attainment, and province. This survey was distributed at three key moments during the COVID-19 pandemic in Flanders, Belgium. The survey was distributed by iVOX, a Belgian market research and online polling agency. Upon completion of the survey, respondents were given a number of virtual points. After collecting a certain amount of points, they were able to purchase gift coupons for restaurant visits, trips, and other activities, from the website of the survey agency. The survey consisted of six themes: socio-demographic characteristics, perceived vulnerability to disease, media- and news consumption, contact with the coronavirus, attitudes towards public health measures, and personality characteristics. In what follows, we highlighted several measures. All data were processed and cleaned through SPSS.

### 2.1 Perceived vulnerability to disease

We used a 15-item self-report instrument to assess perceived vulnerability to disease. Approximately half the items were reverse scored. Participants responded to each item on a 7-point scale with endpoints labelled ‘strongly disagree’ and ‘strongly agree’. This instrument was developed and validated by Duncan, Schaller, and Park [1] and has two subscales: one assesses beliefs about one's own susceptibility to infectious diseases (perceived infectability; eight items; Cronbach's alpha = 0.85), the other emotional discomfort in contexts that connote an especially high potential for pathogen transmission (germ aversion; seven items; Cronbach's alpha = 0.70). After conducting a principal component analysis, the factor scores of both subscales were saved to be used in the analyses.

### 2.2 Socioeconomic And Socio-Psychological Perceptions

We assessed the public's socioeconomic and socio-psychological perceptions regarding the COVID-19 pandemic through three items: if respondents believe that the measures will result in an economic crisis (perception of economic crisis), whether they believe they will be lonely in the coming weeks (loneliness), and whether they will self-quarantine if they feel unwell (solidarity). Participants responded to each item on a 5-point scale with endpoints labelled ‘strongly disagree’ and ‘strongly agree’.

### 2.3 Attitudes towards public health measures

We assessed the public's attitudes towards public health measures installed by the Belgian government through two items, asking if they believe the measures are necessary to protect the population and if they believe that the Belgian government is handling the current crisis well. Again, participants responded to each item on a 5-point scale with endpoints labelled ‘strongly disagree’ and ‘strongly agree’.

### 2.4 Consumption of and opinion on news media

The frequency with which respondents gathered information in the news (public television, commercial television, quality newspapers, tabloids) about the COVID-19 pandemic over the past week was assessed using 5-point scales with endpoints labelled ‘never’ and ‘multiple times a day’. Opinion on news media coverage was assessed by asking respondents’ opinion of the media's coverage of the crisis (1 = media coverage underestimates dangers, 2 = media coverage is accurate, 3 = media coverage overestimates dangers).

### 2.5 Big five personality characteristics

We used a brief measure of the Big Five personality characteristics containing 10 items. Each item contained a personality characteristic, and people were asked to indicate to what extent it applied to them (1 = does not apply at all, 5 = fully apply). The 10 items covered both poles of each personality dimension of the Big Five: extraversion, conscientiousness, agreeableness, openness to experiences, and emotional stability. The version we used was developed by Gosling, Rentfrow and Swann Jr. and “reached adequate levels in terms of: (a) convergence with widely used Big Five measures in self, observer, and peer reports, (b) test–retest reliability, (c) patterns of predicted external correlates, and (d) convergence between self and observer ratings” [2, p. 504].

### 2.6 Brief resilience scale

To measure the degree of resilience in respondents, we used the Brief Resilience Scale developed by Smith et al. [3] and translated into Dutch by Zimmermann [4]. The scale contains six items, and for each item, respondents were asked to indicate to what extent it applies to them (1 = fully disagree, 5 = fully agree). The Dutch version of this scale is a screening instrument with an acceptable reliability, especially concerning the internal consistency of the scale (Cronbach's alpha = 0.74).

## Declaration of Competing Interest

The authors declare that they have no known competing financial interests or personal relationships which have, or could be perceived to have, influenced the work reported in this article.

## Appendix Supplementary materials

Image, application 1

Image, application 2
