# Supplementary material for: Perceptions and opinions on the COVID-19 pandemic in flanders, belgium: Data from a three-wave longitudinal study
Source: Data Brief. 2020 Jul 23;32:106060. doi: 10.1016/j.dib.2020.106060 (PMC7376332; doi:10.1016/j.dib.2020.106060)
Supplement: Supplementary file 2 [file mmc2.pdf]

Socio-demographic characteristics

1. I am …

   1 Male
   2 Female

2. In what year were you born?

3. What is your current marital status?

   1 Unmarried, never been married
   2 Legally or de facto cohabiting
   3 Married
   4 Legally or de facto divorced separated
   5 Widow/widower

4. Is at least one of your living parents (in law) or grandparents 60 years or older?

   1 Yes, at least one biological (grand)parent
   2 Yes, at least one (grand)parent-in-law
   3 No

5. Do you have children?

   1 Yes
   2 No

6. Were you or at least one of your parents born outside Belgium?

   1 Yes
   2 No

Condition: Q6 = 1

7. Please fill in the countries of birth of all persons below.

	Country of birth
Yourself	………………..
Your biological or adoptive mother	………………..
Your biological or adoptive father	………………..

8. Which situation applies to you? Multiple options are possible.

1 I have a full-time job
2 I have a part-time job
3 I am temporarily disabled
4 I am permanently disabled
5 I am a student
6 I am a houseman/housewife
7 I am unemployed
8 I am retired

Condition: Q8 < 5

9. What type of organisation do you currently work for?

1 Central or local government
2 Other organisation in public services (e.g., education, health care)
3 A state-owned company (examples)
4 A private-owned company
5 I am an entrepreneur
6 Other: …………………….
7 Do not know

Condition: Q8< 5

10. Will your company or the company for which you work be closed down in the coming weeks as a result of the measures taken by the Belgian government to contain the coronavirus?

    1 Yes
    2 No

11. Will your company or the company for which you work be closed down in the coming weeks as a result of the measures taken by the Belgian government to contain the coronavirus?

    1 Yes
    2 No
    3 I did not work prior to the corona crisis (unemployment, retired, disability, student, houseman/woman)

Condition: Q8< 5

12. Are you required or invited by your manager to work more from home in the coming weeks?

    1 Yes
    2 No

13. Are you required or invited by your manager to work more from home in the coming weeks?

    1 Yes
    2 No
    3 I did not work prior to the corona crisis (unemployment, retired, disability, student, houseman/woman)

Condition: Q8 = 5

14. Which educational programme are you enrolled in?

1 General secondary education
2 Artistic secondary education
3 Technical secondary education
4 Vocational secondary education
5 Higher non-university education
6 University education

Condition: Q8 < 5 OR Q8 > 5

15. What is your highest educational degree?

1 Uneducated – no diploma or certificate
2 Primary education
3 Lower secondary education
4 Higher secondary education
5 Higher non-university education
6 University education
7 Do not know

16. How difficult or easily does your household make ends meet with the available income of this household (prior to the coronacrisis)?

1 Very difficult
2 Difficult
3 Rather difficult
4 Rather easily
5 Easy
6 Very easy

17. How difficult or easily does your household make ends meet with the available income of this household (considering loss of income due to the coronacrisis)?

1 Very difficult
2 Difficult
3 Rather difficult
4 Rather easily
5 Easy
6 Very easy

18. When it comes to politics, people talk about 'left' and 'right'. Where would you place yourself on the scale below, where 0 stands for far left and 6 for far right?

0<br>Far left	1	2	3<br>Neutral	4	5	6<br>Far right
O	O	O	O	O	O	O

19. For which Flemish party did you vote during the most recent federal elections (May 2019)?

CD&V	O
Groen	O
N-VA	O
Open VLD	O
PVDA	O
sp.a	O
Vlaams Belang	O
Other Flemish party	O
I gave a blank vote	O
I was not yet allowed to vote	O
I did not vote	O
Would rather not say	O

20. In which province do you live?

1 Antwerpen
2 Limburg
3 Oost-Vlaanderen
4 Vlaams-Brabant
5 West-Vlaanderen

21. How would you describe your living environment?

1 A large city
2 The suburbs of a large city
3 A small city
4 A village
5 A farm or house in the countryside
6 Other

1. Perceived vulnerability to disease

22. Indicate to what extent you agree with the following statements.

	0<br>Do not agree at all	1	2	3	4	5	6<br>Fully agree
It really bothers me when people sneeze without covering their mouths.	O	O	O	O	O	O	O
If an illness is ‘going around’, I will get it.	O	O	O	O	O	O	O
I am comfortable sharing a water bottle with a friend.	O	O	O	O	O	O	O
I do not like to write with a pencil someone else has obviously chewed on.	O	O	O	O	O	O	O
My past experiences make me believe I am not likely to get sick even when my friends are sick.	O	O	O	O	O	O	O
I have a history of susceptibility to infectious disease.	O	O	O	O	O	O	O
I prefer to wash my hands pretty soon after shaking someone’s hand.	O	O	O	O	O	O	O
In general, I am very susceptible to colds, flu and other infectious diseases.	O	O	O	O	O	O	O
I dislike wearing used clothes because you do not know what the last person who wore it was like.	O	O	O	O	O	O	O
I am more likely than the people around me to catch an infectious disease.	O	O	O	O	O	O	O
My hands do not feel dirty after touching money.	O	O	O	O	O	O	O

I am unlikely to catch a cold, flu or other illness, even if it is ‘going around’.	O	O	O	O	O	O	O
It does not make me anxious to be around sick people.	O	O	O	O	O	O	O
My immune system protects me from most illnesses that other people get.	O	O	O	O	O	O	O
I avoid using public telephones because of the risk that I may catch something from the previous user.	O	O	O	O	O	O	O

2. (Social) media consumption

23. Through which channels have you sought/received information about the coronavirus in the past week?

	0<br>Never	1	2	3	4<br>Several times a day
Public television (*example*)	O	O	O	O	O
Commercial television (*example*)	O	O	O	O	O
Public radio (*example*)	O	O	O	O	O
Commercial radio (*example*)	O	O	O	O	O
Quality newspapers (*example*)	O	O	O	O	O
Popular newsapapers (*example*)	O	O	O	O	O
Social media of quality newspaper/public broadcaster (*example*)	O	O	O	O	O
Social media of popular newspaper/commercial broadcaster (*example*)	O	O	O	O	O
Internet (via Google, foreign media)	O	O	O	O	O
Face-to-face contact with family/friends/coworkers	O	O	O	O	O
Social media contact with family/friends/coworkers	O	O	O	O	O

24. What is your opinion regarding the media reporting of the coronavirus?

1 Media underestimate the severity of the coronavirus

2 Media provide an accurate representation of the severity of the coronavirus

3 Media overestimate the severity of the coronavirus

25. Which aspects are over- or underestimated according to you?

………

3. Contact with coronavirus

26. For each of the aspects below, please indicate whether or not you are adversely affected by the government measures imposed to contain the coronavirus.

	0<br>No	1	2	3	4<br>Very much
My kids have to stay home.	O	O	O	O	O
I offer extra care to my parents (in-law) or grandparents.	O	O	O	O	O
I offer extra care to local residents.	O	O	O	O	O
I run an extra risk of infection because of my job in the care sector.	O	O	O	O	O
I run the risk of (possible) loss of income.	O	O	O	O	O
I run the risk of (possible) job loss/bankruptcy.	O	O	O	O	O

27. Suppose you have a sore throat. Who or where would you go to first for advice or treatment, now that the coronavirus is in your country?

1 No one
2 Friends or family
3 Pharmacist
4 General practitioner
5 Hospital
6 Internet/online search
7 Medical hotline
8 Other practitioners
9 Do not know

28. Do you believe you have the coronavirus?

1 Yes
2 No

29. Do you believe you have or have had the coronavirus?

1 Yes
2 No

30. Who do you know or believe has or has had the coronavirus? Several answers are possible.

1 Your partner
2 Your child(ren)
3 Your parent(s)
4 Friends/ acquaintances
5 Coworkers
6 Neighbors
7 No one

31. Do you wear a face mask when you go out on the street or go somewhere outside your own home?

1 Yes
2 No

4. Attitudes towards public health measures and government

32. The measures taken by the Belgian government are necessary to protect the population.

1 Fully disagree
2 Disagree
3 Neither agree nor disagree
4 Agree
5 Fully agree

33. The government takes too many measures.

1 Fully disagree
2 Disagree
3 Neither agree nor disagree
4 Agree
5 Fully agree

34. The government takes too few measures.

1 Fully disagree
2 Disagree
3 Neither agree nor disagree
4 Agree
5 Fully agree

35. I fear that the measures - such as the closure of cafes and restaurants - will lead to an economic crisis.

1 Fully disagree

2 Disagree

3 Neither agree nor disagree

4 Agree

5 Fully agree

36. I'm afraid I'll be lonely for the next few weeks.

1 Fully disagree

2 Disagree

3 Neither agree nor disagree

4 Agree

5 Fully agree

37. I've been lonely these past few weeks.

1 Fully disagree

2 Disagree

3 Neither agree nor disagree

4 Agree

5 Fully agree

38. If I feel sick, I'll quarantine myself immediately.

1 Fully disagree

2 Disagree

3 Neither agree nor disagree

4 Agree

5 Fully agree

39. From the moment I felt sick, I immediately quarantined myself.

1 Fully disagree

2 Disagree

3 Neither agree nor disagree

4 Agree

5 Fully agree

40. Persons who have a cough and fever, and who are seen in emergency rooms (but are not sick enough for admission), may be sent home without being examined for the coronavirus.

1 Fully disagree

2 Disagree

3 Neither agree nor disagree
4 Agree
5 Fully agree

41. I think the Belgian government is handling the corona crisis well.

1 Fully disagree
2 Disagree
3 Neither agree nor disagree
4 Agree
5 Fully agree

42. For each of the measures listed below, indicate to what extent you consider them unnecessary or necessary to contain the coronavirus.

	0 Unnecessary	1	2	3	4 Necessary
Non-essential movements are not allowed.	O	O	O	O	O
Keep a minimum distance of 1.5 meters from each other.	O	O	O	O	O
Wash hands thoroughly before and after each contact.	O	O	O	O	O
Suspension of classes at school.	O	O	O	O	O
Closure of non-essential businesses.	O	O	O	O	O
No meetings of more than 2 people who do not live under the same roof.	O	O	O	O	O

43. For each of the relaxations below, indicate to what extent you consider that they have been implemented too quickly or too late.

	0 Too quick	1	2 Right timing	3	4 Too slow
Partial reopening of schools.	O	O	O	O	O

Playing sports together with several people.	O	O	O	O	O
Visit of a maximum of four people.	O	O	O	O	O
Mandatory face mask on public transport.	O	O	O	O	O
Recommended face mask in shops.	O	O	O	O	O
All stores reopened.	O	O	O	O	O

44. I think that a Covid-19 patient in a 'classic household' can be adequately isolated without infecting others (including family members).

1	Fully disagree
2	Disagree
3	Neither agree nor disagree
4	Agree
5	Fully agree

45. For each of the measures below, please indicate the extent to which you follow them.

	0<br>I don't follow this at all	1	2	3	4<br>I follow this perfectly
Non-essential movements are not allowed.	O	O	O	O	O
Keep a minimum distance of 1.5 meters from each other.	O	O	O	O	O
Wash hands thoroughly before and after each contact.	O	O	O	O	O
No meetings of more than 2 people who do not live under the same roof.	O	O	O	O	O

46. For each of the measures below, please indicate the extent to which your child(ren) follow them.

	0<br>They don't follow this at all	1	2	3	4<br>They follow this perfectly
Non-essential movements are not allowed.	O	O	O	O	O
Keep a minimum distance of 1.5 meters from each other.	O	O	O	O	O
Wash hands thoroughly before and after each contact.	O	O	O	O	O
No meetings of more than 2 people who do not live under the same roof.	O	O	O	O	O

47. What did you think was the worst thing about the corona-lockdown?

………

48. Should there be a second wave, and we have to go back into lockdown, what would you do differently?

………

5. Personality characteristics

49. Please indicate to what extent the statements below apply to you.

	1<br>Do not agree at all	2	3	4	5<br>Fully agree
I tend to bounce back quickly after hard times	O	O	O	O	O
I have a hard time making it through stressful events.	O	O	O	O	O
It does not take me long to recover from a stressful event.	O	O	O	O	O
It is hard for me to snap back when something bad happens.	O	O	O	O	O
I usually come through difficult times with little trouble	O	O	O	O	O
I tend to take a long time to get over set-backs in my life.	O	O	O	O	O

50. Below you will find some characteristics that may or may not apply to your personality. Indicate to what extent both characteristics apply to you, even if one applies more than the other.

	1<br>Do not agree at all	2	3	4	5<br>Fully agree
Outgoing, enthusiastic	O	O	O	O	O
Critical, confrontational	O	O	O	O	O
Reliable, disciplined	O	O	O	O	O
Tense, easily in a bad mood	O	O	O	O	O
Open to new experiences, profound	O	O	O	O	O
Reserved, quiet	O	O	O	O	O
Empathic, warm	O	O	O	O	O
Disorganized, sloppy	O	O	O	O	O
Calm, emotionally stable	O	O	O	O	O
Conservative, not creative	O	O	O	O	O
